# Intra-articular CD1c-expressing myeloid dendritic cells from rheumatoid arthritis patients express a unique set of T cell-attracting chemokines and spontaneously induce Th1, Th17 and Th2 cell activity

**DOI:** 10.1186/ar4338

**Published:** 2013-10-20

**Authors:** Frederique M Moret, Cornelis E Hack, Kim MG van der Wurff-Jacobs, Wilco de Jager, Timothy RDJ Radstake, Floris PJG Lafeber, Joel AG van Roon

**Affiliations:** 1 Department of Rheumatology & Clinical Immunology, University Medical Center Utrecht, PO Box 85500, 3508 GA Utrecht, the Netherlands; 2 Center for Molecular and Clinical Immunology, Laboratory of Translational Immunology, University Medical Center Utrecht, PO Box 85500, 3508 GA Utrecht, the Netherlands

## Abstract

**Introduction:**

Myeloid dendritic cells (mDCs) are potent T cell-activating antigen-presenting cells that have been suggested to play a crucial role in the regulation of immune responses in many disease states, including rheumatoid arthritis (RA). Despite this, studies that have reported on the capacity of naturally occurring circulating mDCs to regulate T cell activation in RA are still lacking. This study aimed to evaluate the phenotypic and functional properties of naturally occurring CD1c (BDCA-1)^+^ mDCs from synovial fluid (SF) compared to those from peripheral blood (PB) of RA patients.

**Methods:**

CD1c^+^ mDC numbers and expression of costimulatory molecules were assessed by fluorescence-activated cell sorting (FACS) analysis in SF and PB from RA patients. *Ex vivo* secretion of 45 inflammatory mediators by mDCs from SF and PB of RA patients was determined by multiplex immunoassay. The capacity of mDCs from SF to activate autologous CD4^+^ T cells was measured.

**Results:**

CD1c^+^ mDC numbers were significantly increased in SF versus PB of RA patients (mean 4.7% vs. 0.6%). mDCs from SF showed increased expression of antigen-presenting (human leukocyte antigen (HLA) class II, CD1c) and costimulatory molecules (CD80, CD86 and CD40). Numerous cytokines were equally abundantly produced by mDCs from both PB and SF (including IL-12, IL-23, IL-13, IL-21). SF mDCs secreted higher levels of interferon γ-inducible protein-10 (IP-10), monokine induced by interferon γ (MIG) and, thymus and activation-regulated chemokine (TARC), but lower macrophage-derived chemokine (MDC) levels compared to mDCs from PB. mDCs from SF displayed a strongly increased capacity to induce proliferation of CD4^+^ T cells associated with a strongly augmented IFNγ, IL-17, and IL-4 production.

**Conclusions:**

This study suggests that increased numbers of CD1c^+^ mDCs in SF are involved in the inflammatory cascade intra-articularly by the secretion of specific T cell-attracting chemokines and the activation of self-reactive T cells.

## Introduction

Rheumatoid arthritis (RA) is an autoimmune disorder characterised by persistent joint inflammation resulting in progressive destruction of the joint tissues [1]. CD4^+^ T cells producing T-helper type (Th)-1 (interferon gamma (IFNγ)) and Th17 cytokines (interleukin (IL)-17) [2-5], as well as B cells of the adaptive immune system and macrophages and dendritic cells (DCs) of the innate immune system all contribute to joint inflammation and immunopathology of RA.

DCs are the professional antigen-presenting cells involved in the coordination of adaptive immune responses during infections and against tumour cells. DCs instruct T cells to develop a proper immune response by uptake and presentation of antigens and the provision of costimulatory signals and cytokines. In addition, DCs have the ability to instruct T cells to induce self-tolerance by presenting self-antigens to T cells and subsequent deletion or inactivation of self-reactive T cells [6]. External agents to DCs such as cytokines, tissue-derived factors, pathogen-derived antigens and organic molecules may alter the balance between tolerogenic and immunogenic activity of DCs and induce autoimmune disease [7,8].

Human blood DCs are divided into several phenotypically and functionally different subpopulations including myeloid dendritic cells (mDCs) [9]. mDCs express CD11c and are subdivided into three subsets, of which CD1c^+^ (BDCA-1^+^) mDCs are the most abundant population [10-12]. Since CD1c, apart from mDCs, is only expressed by a subset of B cells, this marker can be used to identify and isolate this unique subset of human mDCs [13,14]. CD1c is a major histocompatibility complex class I-like cell surface glycoprotein that presents lipid and glycolipid self-antigens and nonself-antigens, so CD1c^+^ mDCs can activate restricted lipid antigen-specific T cells [15]. However, these mDCs also have a strong capacity to induce a major histocompatibility complex-dependent antigen-driven allogeneic mixed lymphocyte reaction [11]. CD1c^+^ mDCs in the circulation have been suggested to represent immature DCs that express CD86 and respond to microbial products rather than to inflammatory stimuli (such as tumour necrosis factor alpha) [12]. Recently, CD1c^+^ mDCs were described to have an immunoregulatory function in response to certain microbial triggers [16,17].

Despite the fact that mDCs have been extensively studied in immune disorders in mice and man and that they have been suggested to play an important role in the pathogenesis of RA [18], functional data on naturally occurring mDCs in RA, including those expressing CD1c, are scarce. Previous studies on mDCs in RA were based on CD33/CD14 expression, describing a larger mDC population than the recently defined CD1c^+^ mDCs [19], since CD33 is not only expressed on CD1c^+^ mDCs but also on CD16^+^ and BDCA-3^+^ DC subpopulations [11]. Only a small percentage of CD1c^+^ mDCs express CD14 and the function of these double-positive mDCs is still unknown [20]. In RA, mDCs are increased in the joints as compared with the circulation and express co-stimulatory molecules [21,22]. However, a detailed analysis of the capacity of *ex vivo* cultured CD1c^+^ mDCs from RA patients to produce inflammatory mediators and activate T cells has not been performed.

In the present study, the function of CD1c^+^ mDCs (also referred to as mDCs) from peripheral blood (PB) and synovial fluid (SF) of RA patients was examined. The capacity of mDCs to secrete T cell-differentiating cytokines (including IL-12, IL-33, IL-23), chemokines (including CCL17/thymus and activation-regulated chemokine (TARC), CXCL9/monokine induced by interferon-gamma (MIG), CXCL10/interferon-gamma inducible protein-10 (IP-10)) and proinflammatory cytokines (including IL-1β, IL-6) was studied in combination with their capacity to induce autologous T cell proliferation and cytokine production (IFNγ, IL-17 and IL-4).

## Methods

### Patients

The patients included in this study all met the American College of Rheumatology criteria for RA [23]. Demographic and clinical data of these patients are presented in Table 1. SF and PB samples were collected from the patients. Ethical approval for this study was given by the medical ethical committee of the University Medical Center Utrecht for the collection of patient samples in compliance with the Helsinki Declaration. All patients gave their informed consent.

**Table 1:** Demographic and clinical characteristics of rheumatoid arthritis patients

	**PB**	**SF**	**Paired PB + SF**
Age (years)	51 ± 11	44 ± 6	48 ± 19
Gender (male/female)	4/7	0/5	8/10
Disease duration (years)	15 ± 10	16 ± 2	13 ± 8
Rheumatoid factor (positive/negative)	6/5	2/3	6/9^a^
Erythrocyte sedimentation rate (mm/hour)	16 ± 19	26 ± 22	34 ± 22
C-reactive protein (mg/l)	3 ± 2^b^	14 ± 7	39 ± 61^b^
Treatment (NSAID/corticosteroids/DMARD/biological)	0/0/9/2	0/0/2/3	1/4/5/5^a^

Data presented as mean ± standard deviation or number. DMARD, disease-modifying anti-rheumatic drugs; NSAID, nonsteroidal anti-inflammatory drugs; PB, peripheral blood; SF, synovial fluid. ^a^Three and ^b^eight missing values, respectively.

### Flow cytometry

The number and characteristics of CD1c^+^ mDCs present in mononuclear cell (MNC) fractions of paired PB and SF samples of RA patients were analysed by fluorescence-activated cell sorting (FACS) analysis using a FACS CANTO II flow cytometer (BD Biosciences, San Jose, CA, USA). Cells were stained for CD1c-PE (Biolegend, San Diego, CA, USA), CD19-PERCP-Cy5.5 (BD Biosciences) and CD14 -APC (BD Pharmingen, San Diego, CA, USA) and were identified as CD19-negative and CD1c-positive. Cell surface marker expression on CD1c^+^ mDCs was studied with the following reagents: IgG isotype FITC/PE (Immunotech, Marseille, France), HLA-DR/DP/DQ-FITC (BD Pharmingen), CD80-PE, CD86-PE, CD40-PE and CD19-PERCP-Cy5.5 (all BD Biosciences), and CD1c-Pacific Blue (Biolegend). All data were analysed using FlowJo software (Tree Star, Ashland, OR, USA). To compare mean fluorescence intensity (MFI) values of human leukocyte antigen (HLA) class II, CD80, CD86 and CD40 expression on mDCs from PB and SF, the intensity of autofluorescence assessed using isotype controls from PB mDCs and SF mDCs was subtracted from the MFI of the stainings to reveal true expression differences.

### Cell isolation

MNCs from lithium-heparinised PB and SF were isolated by density centrifugation using Ficoll-Paque Plus (GE Healthcare, Uppsala, Sweden). Prior to MNC isolation, PB or SF was diluted 1:1 with RPMI 1640 medium (Gibco, Life Technologies, New York, USA) containing penicillin (100 U/ml), streptomycin (100 μg/ml), and l-glutamine (2 mM) (all PAA Laboratories, Pasching, Austria). CD1c^+^ mDCs from PB and SF and CD4^+^ T cells from PB were isolated from MNC fractions by magnetic-activated cell sorting using CD1c^+^ (BDCA-1^+^) DC and CD4^+^ T cell isolation kits (Miltenyi Biotec, Bergisch Gladbach, Germany), respectively, according to the manufacturer’s instructions.

### Cell cultures

Cells were cultured in RPMI glutamax (Gibco) supplemented with penicillin (100 U/ml), streptomycin (100 μg/ml), and 10% human AB serum (v/v; GemCell, West Sacramento, CA, USA). Isolated CD4^+^ T cells were seeded in round-bottomed 96-well plates (NUNC, Roskilde, Denmark) at a concentration of 0.5 × 10^6^ cells/ml and stored at 37°C before co-culturing with mDCs. The viable mDCs were counted and equal amounts of live cells were either cultured alone or cocultured with CD4^+^ T cells. To measure cytokine production, isolated mDCs from PB and SF of RA patients were cultured at a concentration of 0.5 × 10^6^ cells/ml for 20 hours at 37°C. Supernatants were harvested and tested for multiple cytokines with multiplex immunoassay. To determine the effect of PB-derived and SF-derived mDCs (≤10,000 cells/well) on autologous CD4^+^ T cells (50,000 cells/well), mDCs derived from PB or SF were added to autologous peripheral CD4^+^ T cells in triplicate at increasing DC:T cell ratios in the absence of additional stimuli. To determine the effect of CD80/86-dependent costimulation in SF mDC/T cell co-cultures, cells were cultured in the presence of CTLA4-Ig (10 μg/ml; Bristol-Myers Squibb, New York, NY, USA). To study whether the observed differences between PB mDCs and SF mDCs were related to mDC maturation, as a control we activated mDCs from PB with thymic stromal lymphopoeitin (TSLP, 20 ng/ml for 20 hours preceding coculture; R&D Systems, Minneapolis, MN, USA). Cells were cocultured in round-bottomed 96-well plates for 6 days and subsequently proliferation and cytokine production were measured. Proliferation was measured by ^3^H-thymidine incorporation assay at the end of the culture period. ^3^H-thymidine (1 μCi/well; PerkinElmer, Waltham, MA, USA) was added during the last 18 hours of the culture period. T cell cytokine production was measured in supernatants of cocultured cells upon short-term restimulation with 500 ng/ml ionomycin and 50 ng/ml phorbol myristate acetate (both Sigma-Aldrich, St Louis, MO, USA) during the last 24 hours of the culture period.

### Cytokine assessment

Cytokines and chemokines in supernatants of cultured mDCs derived from PB and SF were assessed by a multiplex immunoassay as described elsewhere [24]. The cytokines measured and their expression levels are presented in Table 2. T cell cytokines produced by CD4^+^ T cells cocultured with CD1c^+^ mDCs were analysed upon restimulation with ionomycin/phorbol myristate acetate. IFNγ, IL-4 (Invitrogen, Life Technologies, New York, USA) and IL-17 (R&D Systems) were measured by enzyme-linked immunosorbent assay according to the manufacturer’s instructions.

**Table 2:** **Cytokines assessed in supernatants of cultured CD1c**^**+ **^**myeloid dendritic cells derived from PB and SF of rheumatoid arthritis patients**

	**PB (pg/ml)**	**SF (pg/ml)**
**Proinflammatory cytokines**		
IL-1α	409 (150)	344 (343)
IL-1β	258 (65.5)	298 (280)
IL-1RA	2,561 (1,034)	2,769 (3,531)
IL-6	41.0 (13.3)	29.6 (22.6)
IL-6R	324 (100)	343 (199)
IL-18	149 (47.9)	180 (165)
Tumour necrosis factor alpha	99.1 (95.5)	170 (165)
Interferon alpha	153 (69.5)	157 (168)
Thymic stromal lymphopoeitin	<LDL	<LDL
**T cell-differentiating and T cell-activating cytokines**		
IL-12p70	17.6 (11.1)	18.9 (18.5)
IL-23	444 (354)	420 (393)
IL-33	260 (101)	336 (391)
IL-13	857 (387)	1,054 (1,426)
IL-10	112 (49.9)	126 (63.1)
Macrophage migration inhibitory factor	1,872 (704)	2,948 (1,960)
IL-7	25.8 (6.9)	29.2 (29.7)
IL-9	3,087 (1,195)	3,280 (3,758)
IL-15	21.7 (8.7)	29.4 (27.3)
IL-21	5,114 (1,834)	6,343 (4,710)
IL-22	19.2 (6.1)	29.0 (27.3)
IL-25	1,140 (526)	1,239 (1,310)
**Chemokines**		
IL-16	239 (101)	633 (581)^†^
CCL2/MCP1	29.7 (18.5)	24.6 (25.5)
CCL3/MIP1α	1,268 (1,847)	2,776 (3,954)
CCL5/RANTES	<LDL	<LDL
CCL17/TARC	1.2 (0.5)	26.4 (23.6)**
CCL19/MIP3β	28.4 (11.5)	57.5 (51.5)
CCL22/MDC	4,397 (1,627)	2,456 (1,023)*
CXCL9/MIG	23.9 (16.1)	90.4 (67.9)**
CXCL10/IP-10	54.0 (70.3)	247 (310)*
**Growth factors/others**		
Oncostatin M	28.0 (21.7)	23.5 (17.8)
Fibroblast growth factor basic	4,046 (1,647)	4,777 (5,559)
Nerve growth factor	63.0 (23.2)	76.7 (90.2)
Hepatocyte growth factor	118 (46.8)	151 (143)
Granulocyte–macrophage colony-stimulating factor	650 (283)	827 (859)
Macrophage colony-stimulating factor	484 (188)	571 (730)
Vascular endothelial growth factor	191 (110)	202 (125)
IL-11	49.6 (20.1)	53.6 (63.9)
Soluble intracellular adhesion molecule-1	34,067 (139,11)	37,260 (46,225)
Osteopontin	11,192 (4,471)	15,528 (10,471)
Matrix metalloproteinase-8	2,237 (1,594)	2,742 (4,634)
Tissue inhibitor of metalloproteinases-1	2,689 (1,126)	2,455 (2,169)
Cathepsin B	4,204 (1,554)	6,283 (4,948)
Cathepsin L	215 (101)	594 (731)
Cathepsin S	133 (55.3)	159 (130)

Data presented as mean (standard deviation). IP-10, interferon-gamma inducible protein-10; LDL, lower detection limit; MCP-1, monocyte chemotactic protein-1; MDC, macrophage-derived chemokine; MIG, monokine induced by interferon-gamma; MIP, macrophage inflammatory protein; PB, peripheral blood; RANTES, regulated upon activation, normally T cell-expressed, and presumably secreted; SF, synovial fluid; TARC, thymus and activation-regulated chemokine; IL, interleukin. *P <0.05; **P <0.01; ^†^*P* = 0.10.

### Statistical analysis

Paired-sample evaluation was performed using the nonparametric Wilcoxon signed-rank test. Unpaired data analysis was performed using the nonparametric Mann–Whitney *U* test. Data analysis was performed using SPSS software (IBM, Armonk, NY, USA). Data were considered statistically significant at *P* <0.05.

## Results

### CD1c^+^ mDCs have an increased frequency and activated phenotype in RA synovial fluid

**Figure 1:**
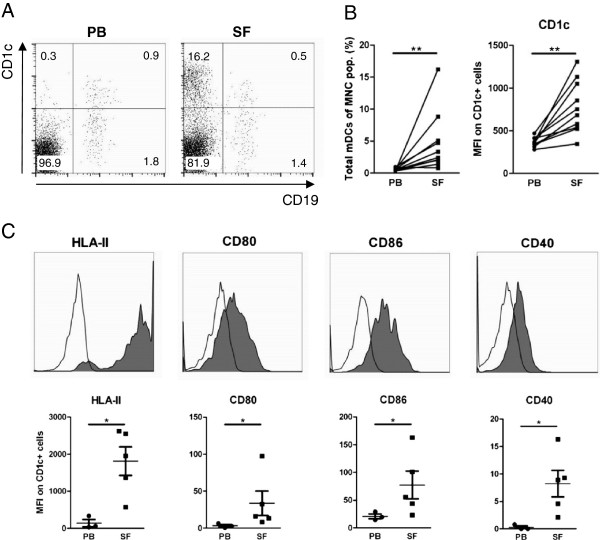
**CD1c**^**+ **^**myeloid dendritic cells are abundantly present in joints of rheumatoid arthritis patients and express increased levels of antigen-presenting and costimulatory molecules. (A)** Representative fluorescence-activated cell sorting (FACS) dot plot of CD1c-expressing myeloid dendritic cells (mDCs) and CD19^+^ cells in the peripheral blood (PB) and synovial fluid (SF) of a rheumatoid arthritis (RA) patient. **(B)** mDC numbers are increased in SF versus PB of RA patients (*n* = 10), and SF mDCs express higher CD1c levels. Percentages (%) of the total mononuclear cell (MNC) population and mean fluorescent intensity (MFI) of CD1c expression are given. **(C)** mDCs derived from SF (*n* = 5) express enhanced levels of antigen-presenting (human leukocyte antigen class II (HLA-II)) and costimulatory molecules (CD80, CD86 and CD40) as compared with PB-derived mDCs (*n* = 3). Representative histograms of isotype control (open) and HLA-II, CD80, CD86 and CD40 (filled) expression and mean are shown (MFI corrected for isotype fluorescence). Statistically significant differences of *P <0.05 and **P <0.01.

mDCs were characterised by the expression of CD1c and the absence of CD19 in MNC fractions of paired PB and SF samples (*n* = 10) of RA patients (representative dot plot; Figure 1A). The number of mDCs, determined as a percentage of total MNCs, was significantly increased in SF versus PB (mean ± standard error of the mean: 4.7 ± 1.5% vs. 0.6 ± 0.1%, respectively, *P* <0.01) as well as the CD1c expression (MFI: 778 ± 97 vs. 372 ± 18, respectively, *P* <0.01; Figure 1B). Cell surface markers on mDCs derived from PB and SF of RA patients were also studied (*n* = 3 and *n* = 5, respectively). mDCs derived from SF compared with those from PB expressed increased levels of antigen-presenting molecules HLA class II and costimulatory molecules CD80, CD86 and CD40 (representative histograms and MFI; Figure 1C). In addition to the MFI, the percentage of SF mDCs expressing all these activation markers was also significantly increased compared with PB mDCs (mean ± standard error of the mean % positive cells in PB vs. SF, respectively: HLA class II, 39 ± 10% vs. 90 ± 3%, *P* <0.05; CD80, 4 ± 1% vs. 35 ± 10%, *P* <0.05; CD86, 30 ± 7% vs. 63 ± 10%, *P* = 0.07; CD40, 0.2 ± 0% vs. 11 ± 3%, *P* <0.05).

### Synovial fluid-derived CD1c^+^ mDCs from RA patients secrete higher levels of specific cytokines compared with peripheral blood mDCs

**Figure 2:**
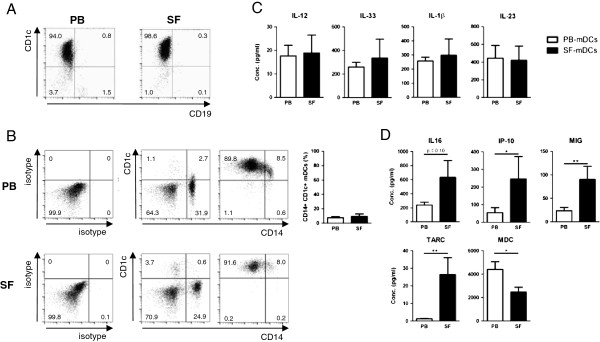
**CD1c**^**+ **^**myeloid dendritic cells from synovial fluid of rheumatoid arthritis patients produce increased chemokine levels but equal amounts of T cell-differentiating cytokines compared with those from peripheral blood. (A)** Representative dot plot of isolated CD1c-expressing myeloid dendritic cells (mDCs) from peripheral blood (PB) and synovial fluid (SF) of a rheumatoid arthritis (RA) patient. **(B)** Representative dot plots of isotype (left), CD1c and CD14 expression on CD19^–^ mononuclear cells (middle) and on isolated mDCs (right plot) from PB and SF. A small percentage of mDCs from PB and SF (*n* = 6, paired samples) expresses CD14 (bar graph). **(C)** PB mDCs and SF mDCs (*n* = 6) produced comparable levels of T-helper type (Th)-1, Th17 and Th2-differentiating cytokines interleukin (IL)-12, IL-23, IL-33. **(D)** Production of several chemokines by SF mDCs was significantly upregulated and macrophage-derived chemokine (MDC) significantly downregulated as compared with PB mDCs. Apart from the T cell-differentiating cytokines, only inflammatory mediators that showed *P* ≤0.10 are shown. Statistically significant differences of *P <0.05 and **P <0.01. IP-10, interferon-gamma inducible protein-10; MIG, monokine induced by interferon-gamma; TARC, thymus and activation-regulated chemokine.

To study the functional properties of CD1c^+^ mDCs these were isolated from PB MNCs and SF MNCs of RA patients (*n* = 6; representative dot plot, Figure 2A). A small subset of CD1c^+^ mDCs has been described to express CD14. Therefore, the percentage of CD14-expresssing cells was assessed in CD19^–^ MNC fractions and in isolated CD1c^+^ cells. Only a small proportion of isolated CD1c^+^ cells expressed CD14, to a similar extent on CD1c^+^ mDCs from PB and SF (*n* = 6; representative dot plots and mean of paired PB and SF samples, Figure 2B).

Isolated CD1c^+^ cells were cultured for 20 hours in the absence of stimuli and cytokine production was assessed (Table 2). Production of T cell-differentiating cytokines IL-12p70, IL-33, IL-1β, and IL-23 by mDCs from SF was not significantly different from that of PB mDCs (all *P* >0.10; Figure 2C). The secretion of the chemoattractive mediators IP-10, MIG, and TARC by SF mDCs was significantly increased as compared with PB mDCs. Macrophage-derived chemokine (MDC) production was significantly decreased by SF mDCs compared with PB mDCs (Figure 2D). The production of the T cell attractant IL-16 by SF mDCs was also elevated compared with PB mDCs, although this did not reach statistical significance (*P* = 0.10; Figure 2D).

Inflammatory mediators that were under the detection limit included RANTES (regulated upon activation, normally T cell-expressed, and presumably secreted) and TSLP. All other mediators were secreted in equal amounts by PB and SF mDCs (all *P* >0.10) (Table 2).

### Intra-articular CD1c^+^ mDCs spontaneously induce proliferation and cytokine secretion of autologous CD4^+^ T cells in RA patients

**Figure 3:**
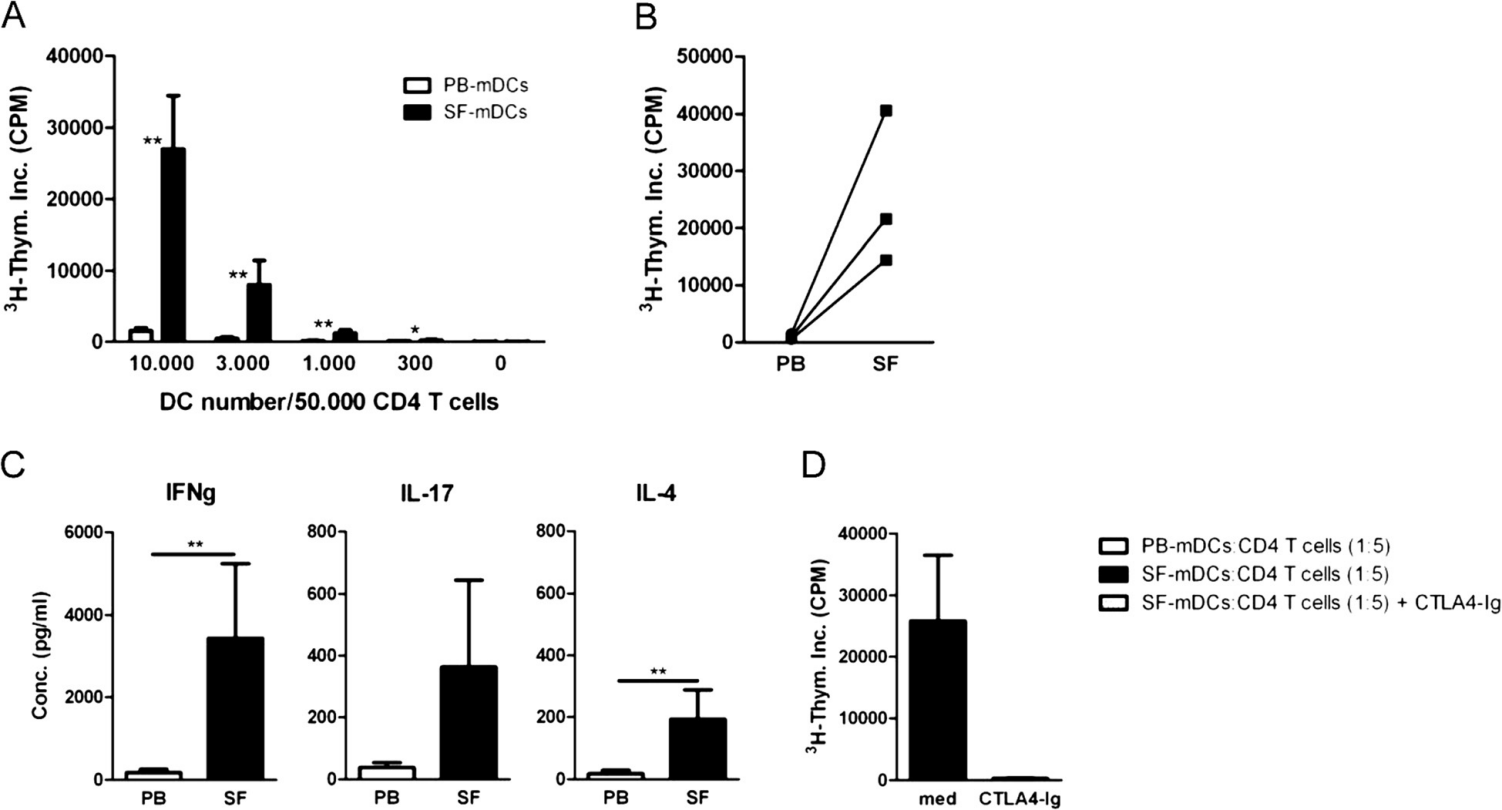
**Intra-articular CD1c**^**+ **^**myeloid dendritic cells induce strong proliferation of CD4**^**+ **^**T cells associated with a strong increase in proinflammatory T cell cytokine production. (A)** Inflammatory myeloid dendritic cells (mDCs) from synovial fluid (SF) when added to autologous CD4^+^ T cells induced a strong spontaneous proliferation of CD4^+^ T cells of rheumatoid arthritis (RA) patients (*n* = 5), significantly higher compared with mDCs from peripheral blood (PB; *n* = 11). **(B)** Paired analysis of mDCs from PB and SF from the same donors (*n* = 3, 10,000 DCs/50,000 CD4^+^ T cells) showed a similar robust induction of autologous T cell proliferation by SF mDCs. **(C)** mDCs from SF (*n* = 5) induced a strong production of proinflammatory cytokines (interferon gamma (IFNγ), interleukin (IL)-17 and IL-4) compared with PB mDCs (*n* = 11). **(D)** SF mDC-induced T cell proliferation was strongly dependent on CD80/86 costimulation as it was completely blocked in the presence of CTLA4-Ig. Statistically significant differences of *P <0.05 and **P <0.01. CPM, counts per minute.

Since mDCs from SF express increased levels of antigen-presenting and costimulatory molecules and produced abundant amounts of T cell-differentiating cytokines as well as increased levels of several inflammatory mediators compared with mDCs from PB, we investigated the capacity of PB-derived and SF-derived mDCs (*n* = 11 and *n* = 5, respectively) to activate autologous CD4^+^ T cells. CD4^+^ T cells co-cultured with SF mDCs showed a robust increase in the induction of T cell proliferation compared with PB mDCs (PB mDCs vs. SF mDCs:CD4 T cells (1:5), 1,503 ± 443 vs. 26,935 ± 7,543 counts/minute, *P* <0.01, respectively; Figure 3A). Similar increased T cell activation was observed when paired PB and SF mDCs from the same donor were analysed (Figure 3B). The increase in the induction of T cell proliferation was also associated with a robust increase in the production of T cell cytokines, measured after restimulation with ionomycin/phorbol myristate acetate. The production of IFNγ, IL-17 and IL-4 was higher by SF mDCs versus PB mDCs (Figure 3C). To test whether enhanced SF mDC-induced T cell activation was dependent on CD80/86 upregulation, CD80/86 costimulation was blocked by CTLA4-Ig. SF mDC-induced T cell activation was completely blocked by CTLA4-Ig (Figure 3D). To study whether the observed differences between PB and SF mDCs were related to DC maturation, as a control, we activated mDCs from PB with TSLP, previously shown to activate CD11c^+^ DCs and implicated to play a proinflammatory role in RA [25,26]. TSLP-stimulated CD1c^+^ mDCs induced robust proliferation of autologous CD4^+^ T cells (unstimulated vs. TSLP-stimulated mDCs:CD4 T cells (1:5), proliferation: from 1,000 ± 284 to 14,206 ± 2,426 counts/minute, *P* = 0.01, respectively; *n* = 8 from PB of healthy controls).

## Discussion

Hitherto, functional data on CD1c^+^ (BDCA-1^+^) mDCs from RA patients are scarce. Here we demonstrate that in RA patients naturally occurring CD1c^+^ mDCs are present in higher frequency in SF and that these mDCs have an activated phenotype and secrete increased levels of a unique set of chemokines in comparison with mDCs from PB. To our knowledge this is also the first study that reports CD1c^+^ mDCs from SF of RA patients to compellingly cause autologous T cell activation.

The majority of T cells present in SF of RA patients were previously shown to express the CXC chemokine receptor 3 (CXCR3), with a higher percentage of T cells expressing CXCR3 in SF than in PB of RA patients [27,28]. CXCR3 is mainly expressed on Th1 cells and binds to its ligands CXCL10/IP-10 and CXCL9/MIG [29,30]. Elevated IP-10 and MIG levels are observed in SF of RA patients compared with control SF from patients with osteoarthritis or traumatic joint injury [27], and the present study shows that *in vivo*-activated CD1c^+^ mDCs from RA joints secrete high levels of IP-10 and MIG. Furthermore, these mDCs secrete higher CCL17/TARC levels compared with mDCs derived from PB. TARC is a selective ligand for the CC chemokine receptor 4 (CCR4) that is expressed by Th2 cells [29,30], Th17 cells [31], and regulatory T cells [32], suggesting that TARC contributes to attraction of these T cells into the inflamed joints. Secretion of CCL22/MDC by SF mDCs, another CCR4 ligand, was significantly lower compared with that by PB mDCs. To what extent this differential expression of CCR4 ligands influences the chemotaxis of Th2, Th17 and regulatory T cells remains to be demonstrated. Recently, it was demonstrated that MDC levels in SF of RA patients are elevated compared with osteoarthritis patients [33]. This may indicate that other cell types could contribute to these enhanced levels. Alternatively, the increased number of CD1c^+^ mDCs (~5-fold higher) in the joint compared with the circulation of RA patients could contribute to elevated intra-articular levels.

Apart from enhancing immune activation, DCs induced under different conditions can also induce tolerance – by increasing regulatory T cell activity, amongst other effects [7,8]. Our data demonstrate that, in line with the enhanced expression of antigen-presenting and costimulatory molecules, SF-derived CD1c^+^ mDCs spontaneously increased proliferation and Th1, Th17 and Th2 cell cytokine production of autologous CD4^+^ T cells. Although we did not measure regulatory T cell function, our data demonstrate that mDCs from SF overrule suppression by regulatory T cells and indicate that *in vivo*-activated CD1c^+^ mDCs from the joint have a strong capacity to induce T cell expansion of infiltrating T cells without strongly skewing the T cell balance. In line with this observation, SF and PB mDCs secreted equal amounts of T cell-differentiating cytokines. Since in RA joints a predominance of Th1 and Th17 activity is observed, our data suggest that in joints of RA patients further Th1 and Th17 cell skewing is facilitated by local inflammatory mediators (for example, IL-12) produced, for example, by macrophages.

Recently, Frankenberger and colleagues showed via gene expression analysis that CD14^+^^+^CD16^-^ as well as CD16^+^ monocytes are both distinct from the CD1c^+^ blood DCs [34]. In the present study, CD14-expressing cells that lacked CD1c were not present or were hardly present in the isolated DC fractions (~0.5%); however, a small percentage of the CD1c^+^ mDCs coexpressed CD14, similar to previous reports for healthy controls [20]. Our data demonstrate that activated mDCs from SF of RA patients show a robust homogenic upregulation of HLA class II, CD80, CD86, CD40 and CD1c compared with their circulating counterparts. This corresponds to the robust T cell stimulatory capacity of these SF mDCs and contradicts the assumption that a subpopulation of CD14^+^ cells is responsible for the observed effects. In addition, the percentages of CD14^+^CD1c^+^ mDCs between PB and SF did not differ. These latter suggestions are in line with the much lower T cell stimulatory capacity of total CD14^+^ monocytes, either isolated from SF or from PB (unpublished data). Recently, however, CD1c^+^ DCs expressing CD14 were suggested to represent human inflammatory DCs, present in inflammatory environments such as ascites and RA joints, and potent inducers of Th17 cells [35]. Although individual data from RA SF cannot be deduced from this latter study, CD14 seems to be expressed at higher levels on inflammatory CD1c^+^ mDCs versus blood CD1c^+^ mDCs [35]. Differences in this latter study and the present study, showing low and comparable percentages of CD14^+^CD1c^+^ mDCs between PB and SF, might be related to the site of inflammation studied (SF vs. ascites), but this remains to be established.

Although a wide range of triggers might activate CD1c^+^ mDCs, the predominant triggers that activate these cells in RA joints remain to be demonstrated [36]. Jongbloed and colleagues investigated the capacity of SF mDCs to respond to toll-like receptor-2 agonism. As compared with healthy PB mDCs, mDCs from the joints of RA patients produced equal amounts of tumour necrosis factor alpha and increased amounts of IL-10 in response to activation by *Staphylococcus aureus* peptidoglycan [22]. Based on this study of toll-like receptor triggering, mDCs from the joints of RA patients were suggested not to be activated and to display a semi-mature phenotype [22]. In line with these data the present study also failed to detect any robust differences between PB and SF mDCs in secretion of cytokines (including IL-1, IL-6, tumour necrosis factor alpha, IFNα) typically induced upon toll-like receptor-2 or other toll-like receptor triggering. However, the present data clearly reveal an activated status of SF mDCs and a powerful stimulation of T cells by these SF mDCs, which suggests that stimuli other than toll-like receptor ligands contribute to the enhanced activity of these cells *in vivo,* associated with a unique set of secreted chemokines. Recently, increased levels of TSLP in SF of RA patients versus osteoarthritis patients were documented [37,38]. TSLP strongly induced TARC production by mDCs from PB and SF of RA patients [38]. Furthermore, mDCs from the peripheral blood, activated by TSLP, showed phenotypical and functional similarities with mDCs derived from the joints of RA patients, suggesting that TSLP might be an important trigger for mDC activation in RA joints [38].

## Conclusions

There is clear evidence that multiple subsets of DCs exist, which display specialised functions although the tissue microenvironment and the stage of maturation can influence their phenotype and function. The present study suggests that accumulating CD1c^+^ mDCs in the joints of RA patients importantly contribute to inflammation by inducing secretion of a unique set of T cell-attracting chemokines and by spontaneously activating CD4^+^ T cells to proliferate and secrete proinflammatory cytokines. The identification of the factors that control the capacity of mDCs to elicit Th1, Th17 and Th2 development in RA is critical to envisage new approaches to manipulate the immune system to the benefit of these patients.

## Abbreviations

DC: Dendritic cell; HLA: Human leukocyte antigen; IFNγ: Interferon gamma; IL: Interleukin; IP-10: Interferon-gamma inducible protein-10; MDC: macrophage-derived chemokine; mDC: Myeloid dendritic cell; MIG: Monokine induced by interferon gamma; MFI: Mean fluorescence intensity; MNC: Mononuclear cell; PB: Peripheral blood; RA: Rheumatoid arthritis; SF: Synovial fluid; TARC: Thymus and activation-regulated chemokine; Th: T-helper type; TSLP: Thymic stromal lymphopoeitin.

## Competing interests

The authors declare that they have no competing interests.

## Authors’ contributions

FMM participated in the study design, acquisition of data, analysis and interpretation of data and writing of the manuscript. CEH contributed to the study design, interpretation of data and writing of the manuscript. KMGvdW-J and WdJ contributed to the acquisition of data. TRDJR and FPJGL were involved in the interpretation of data and writing of the manuscript. JAGvR contributed to the study design, analysis and interpretation of data and writing of the manuscript. All authors read and approved the final manuscript for publication.
